# Protocol for isolation and characterization of small extracellular vesicles from human menstrual fluid using size-exclusion chromatography

**DOI:** 10.1016/j.xpro.2026.104573

**Published:** 2026-05-18

**Authors:** Yiqun Tang, Ruihan Zhou, Caroline Frisendahl, Kristina Gemzell-Danielsson

**Affiliations:** 1 WHO Collaborating Centre, Division for Neonatology, Obstetrics, Gynecology and Reproductive Health, Department of Women’s and Children’s Health, Karolinska Institutet, Biomedium A4, Solnavägen 9, 171 65 Stockholm, Sweden; 2 Division of Gynecology and Reproductive Medicine, Karolinska University Hospital, Stockholm, Sweden; 3 Pediatric Oncology and Pediatric Surgery, Department of Women’s and Children’s Health, Karolinska Institutet, Biomedium A4, Solnavägen 9, 171 65 Stockholm, Sweden

**Keywords:** Cell isolation, Health Sciences, Protein expression and purification

## Abstract

Human menstrual fluid (MF) is rich in extracellular vesicles originating from peripheral blood components and local endometrial and immune cells. Here, we present a protocol to obtain MF-derived small extracellular vesicles (MF-sEVs) using dilution, low-speed centrifugation, filtration, and size-exclusion chromatography. We describe steps for characterizing pooled fractions by transmission electron microscopy (TEM), nanoparticle tracking analysis (NTA), and western blotting. This workflow yields high-quality MF-sEVs suitable for reproducible omics and functional assays, enabling robust biomarker discovery and therapeutic exploration in uterine disorders.

## Highlights

• Steps for isolating MF-sEVs by size-exclusion chromatography
• Instructions for dilution, centrifugation, and filtration of menstrual fluid
• Guidance on fraction collection by size-exclusion chromatography
• Procedures for MF-sEVs characterization by TEM, NTA, and western blotting

## Abstract (editor highlights)

Publisher’s note: Undertaking any experimental protocol requires adherence to local institutional guidelines for laboratory safety and ethics.

## Graphical abstract

**Figure:**
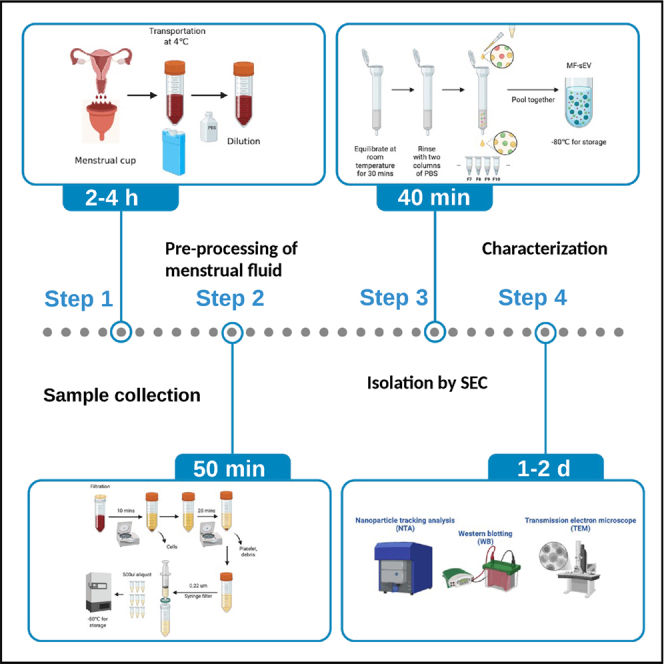

## Before you begin

### Innovation

This protocol provides an optimized workflow for isolating small extracellular vesicles (sEVs) from human menstrual fluid (MF), a non-invasive sample with high cellular, protein, and RNA complexity. It incorporates dilution, low-speed centrifugation, and filtration steps tailored to MF, followed by size-exclusion chromatography for consistent fractionation. These steps improve sample clarity and reduce contamination from debris and protein aggregates while preserving vesicle integrity. The protocol also outlines standardized characterization using transmission electron microscopy, nanoparticle tracking analysis, and western blotting, supporting compatibility with downstream omics and functional assays. Overall, this workflow enables reproducible isolation of MF-sEVs for studies of uterine biology and disease.

### Materials to prepare in advance

**Timing: Approximately 30 min**

This section describes the preparation of materials and solutions required prior to small extracellular vesicle isolation from menstrual fluid using qEVoriginal Gen 2 70 nm columns (Izon Science Ltd, Christchurch, New Zealand). All solutions should be prepared under sterile conditions in a clean biosafety cabinet.

1. Menstrual cup.
  a. Lunette, model 2 – available in large, medium, and small sizes.
  b. Used for self-collection of menstrual fluid by participants following manufacturer’s instructions under proper guidance.
2. Particle-free Phosphate-Buffered Saline (DPBS).
  a. Assemble the filtration system by connecting a 0.22 μm 500 mL filter unit to the pump using sterile tubing.
  b. Pre-rinse the filter membrane with 10–20 mL sterile DPBS and discard the flow-through to minimize nonspecific absorption of extracellular vesicles to the membrane.
  c. Pour the prepared DPBS into the upper reservoir of the filter unit.
  d. Start the pump and allow the solution to pass through the membrane into the sterile collection bottle.
  e. Store the particle-free DPBS at room temperature for future use.
3. Particle-free 20% ethanol.
  a. Assemble the filtration system by connecting a 0.22 μm 500 mL filter unit to the pump using sterile tubing.
  b. Prepare 20% (v/v) ethanol by mixing 100 mL absolute ethanol with 400 mL sterile distilled water.
  c. Pre-rinse the filter membrane with 10–20 mL sterile DPBS and discard the flow-through.
  d. Pour the prepared 20% ethanol into the upper reservoir of the filter unit.
  e. Start the pump to allow the solution to pass through the membrane into the sterile collection bottle.
  f. Store the particle-free 20% ethanol at room temperature for future use.

***Note:*** The 20% ethanol solution is used for cleaning and regeneration of IZON qEVoriginal columns immediately after EV isolation according to qEVoriginal Gen 2 user manual.

4. Particle-free 0.5 M NaOH solution.
  a. Weigh 20 g of NaOH pellets and dissolve them gradually in approximately 800 mL of distilled water while stirring.
    **CRITICAL:** The dissolution of NaOH is strongly exothermic. Add the pellets slowly in small portions, with continuous stirring, and allow the solution to cool between additions to avoid overheating or splashing.
  b. After the pellets are completely dissolved and the solution has cooled to room temperature, adjust the final volume to 1 L with distilled water.
  c. Assemble the filtration system by connecting a 0.22 μm 500 mL filter unit to the pump using sterile tubing. Pour the prepared 0.5 M NaOH solution into the upper reservoir of the filter unit. Start the pump, allowing the solution to pass through the membrane into the sterile collection bottle.
  d. Store the particle-free 0.5 M NaOH solution at room temperature for future use.
    ***Note:*** The 0.5 M NaOH solution is used for cleaning and regeneration of IZON qEVoriginal columns immediately after EV isolation according to qEVoriginal Gen 2 user manual.

### Institutional permissions

This study was reviewed and approved by the Swedish Ethical Review Authority (original approval: Dnr 2021-05157; amendments: Dnr 2022-01635-02 and 2025-07599-02) to include non-invasive menstrual fluid collection from healthy volunteers. The collection of menstrual fluid was performed using anonymized participant information. All participants have received information about the study before enrollment. Informed consent was obtained from all participants prior to enrollment. Inclusion criteria were age 18–52 years, non-smoker, at least one spontaneous pregnancy, regular menstrual cycles with a cycle length of 21–35 days and menstruation of 3–7 days. Exclusion criteria included BMI > 35, major systemic illnesses (autoimmune, endocrine disorders), ongoing malignancies, and gynecological disorders. All aspects of volunteer recruitment, sample collection, and data handling followed approved protocols and institutional guidelines.

## Key resources table

**Table:** 

REAGENT or RESOURCE	SOURCE	IDENTIFIER
**Antibodies**		

Anti-CD9 (1:1000)	SBI	EXOAB-CD9A-1
Anti-CD81 (1:500 - 1:2000)	Immunoway	YT5394
Anti-CD63(1:1000)	SBI	EXOAB-CD63A-1
Anti-Calnexin (1:500 - 1:1000)	Immunoway	YT0613
Exosome validated secondary antibody (Goat anti-Rabbit HRP (1:20000)	SBI	EXOAB

**Chemicals, peptides, and recombinant proteins**		

NanoStandards – 0.100 μm	Microspheres	750090–03
Sodium Hydroxide, Pellets	EMD Millipore Corp.	567530-250GM
Absolute ethanol	Histolab Products AB	64-17-5
DPBS, no calcium, no magnesium	Thermo Fisher Scientific	21600010
UltraPure™ 0.5 M EDTA, pH 8.0	Merck KGaA- Sigma-Aldrich	15575020

**Other**		

qEV (70nm) size exclusion chromatography columns (Original Column Gen 2)	IZON	C20-70
IZON qEV Rack (qEVoriginal)	IZON	QR1
Nanoparticle Tracking Analyzer	ZetaView	PMX-230-7
0.22 μm filter	Merck Millipore	S2GPU05RE
Lunette, model 2	Lunette Global	434425–0070
Talos 120 TEM	Thermo Fisher Scientific	–
ROTINA 420 R Centrifuge	Hettich	4706–01
500 ml Filter System, 0.22 μm, General Purpose, Nonpyrogenic Polystyrene	Corning	430756
WOB-L PRES/VAS DRY PUMP 2, Model No.2522Z-02 A	WELCH	12150001289
Vivaspin® 2 Centrifugal Concentrator(10 kDa MWCO)	Sartorius	VS02V2
Cell strainer, 40 μm	FALCON, Corning	352340
Cell strainer, 100 μm	FALCON, Corning	352360
Maxymum Recovery® Snaplock Microcentrifuge Tube	Axygen®	MCT-150-L-C

## Step-by-step method details

### Collection of menstrual fluid samples

**Timing: Approximately 2–5 h**

This step describes the standardized procedure for collecting menstrual fluid for subsequent isolation of small extracellular vesicles (sEVs).

1. On the second day of the menstrual cycle, when flow is heaviest, insert a menstrual cup (Lunette, model 2; Lunette Global, SE; Cat#434425-0070) for approximately **2∼4 h** during daytime.
2. After removal, carefully transfer **2∼10 mL** of collected menstrual fluid into a pre-cooled 50 mL sterile Falcon tube (ThermoFisher Scientific, #339652).

***Note:*** The minimum of **2 mL** menstrual fluid is required. Collections yielding <2 mL should be regarded as insufficient for further processing.

3. Immediately place the collection tube in an ice bag for transportation. Samples should reach the laboratory within **1 h** of collection and be maintained at **4°C**.
4. Upon arrival at the laboratory, record sample metadata (collection time, participant code, sample volume).
5. Proceed to the isolation of menstrual fluid serum immediately as outlined in the next section.

**CRITICAL:** Maintain samples at **4°C** during transportation and handling to prevent EV degradation.

### Isolation of serum from menstrual fluid

**Timing: Approximately 40 min**

This step describes the removal of cells, platelets, and large debris from menstrual fluid to obtain a clarified, cell-free menstrual serum suitable for small extracellular vesicle isolation.

6. Upon arrival at the laboratory, dilute the menstrual fluid with an equal volume of particle-free DPBS. Gently mix by inversion to avoid bubble formation.

***Note:*** Depending on downstream applications, users may consider including protease inhibitors during early processing steps to minimize protein degradation.

7. Pre-wet a **100** **μm** cell strainer (Falcon, Corning, #352360) with particle-free DPBS, pass the diluted sample through into a new 50 mL conical tube.
8. Centrifuge at **400 × g** for **10 min** at **4°C** to remove intact cells and big debris. Carefully collect the supernatant without disturbing the pellets.
9. Centrifuge the filtrate at **2,000 × g** for **20 min** at 4°C.
  a. Transfer the clarified supernatant to a new sterile 50 mL Falcon tube.
10. Pass the supernatant through a **0.22** **μm** syringe filter (Merck Millipore Ltd., Cat# SLGP033RS) to obtain sterile cell-free menstrual serum.

***Note:*** The 0.22 μm filtration step is included to remove residual debris and aggregates that may interfere with downstream size-exclusion chromatography performance. However, filtration may also result in partial loss of vesicles due to membrane retention or clogging, particularly for viscous samples. Users may consider evaluating particle recovery (e.g. by NTA before and after filtration) and adjusting the filtration strategy depending on sample characteristics and whether purity or yield is prioritized.

11. Dispense the filtered serum into **500** **μL** aliquots using sterile low binding tubes (Axygen, MCT-150-L-C). Proceed to sEV isolation immediately or store the aliquots at −80°C until use for EV isolation. Avoid repeated freeze–thaw cycles.

***Note:*** The menstrual blood serum can be stored at *−*80°C for up to half a year prior to EV isolation without significant loss of EV integrity. Samples should be thawed on ice before use.

### Isolation of menstrual fluid-derived sEVs by size-exclusion chromatography

**Figure 1:**
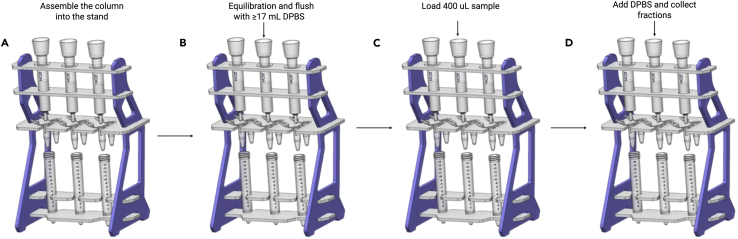
Schematic illustration of MF-sEVs isolation using a qEVoriginal 70 nm Gen 2 column mounted on a stand (A) qEVoriginal rack assembled correctly. (B) Columns are equilibrated with particle-free DPBS. (C) Serum samples are loaded onto the loading frit. (D) Fractions are collected sequentially for sEVs isolation. Created with BioRender.com.

This step describes the isolation of small extracellular vesicles (sEVs) from clarified menstrual serum using qEVoriginal 70 nm (Gen 2) columns (Izon Science Ltd, Christchurch, New Zealand) (Figure 1).

Size-exclusion chromatography (SEC) separates particles according to their hydrodynamic size, allowing larger vesicles to elute earlier, while smaller proteins and soluble biomolecules penetrate the pores of the resin and elute later.^1^ This principle enables efficient separation of nanosized vesicles from abundant soluble proteins in biological fluids.

### Assembly of qEVoriginal rack and column setup

**Timing: Approximately 45 min**

12. Rack assembly.
  a. Place the base platform on a level bench surface.
  b. Insert the two vertical support arms into the base until securely fixed.
  c. Attach the top plate by aligning the side slots and pressing downward until the plate locks into position.
  d. Verify that the rack is stable and upright before inserting the column.

***Note:*** Refer to the manufacturer’s assembly manual: “qEVoriginal Rack Assembly Instructions” Izon Science.

13. Column equilibration.
  a. Insert the column vertically into the rack holder with the narrow luer outlet facing downward.
  b. Equilibrate the column and the sample buffer to be within the operational temperature range of 18°C–24°C for **30 min.**

***Note:*** Do not remove the caps until the column has reached operational temperature.

14. Column flushing.
  a. Remove the bottom luer cap to initiate gravity flow.
  b. Allow the storage buffer to start running through the column.
  c. Flush the column with at least **17 mL** particle-free DPBS to the top reservoir and allow it to pass completely through the column by gravity.
  d. Collect the effluent into a waste container.

**CRITICAL:** If the column will not be used immediately, close the bottom luer cap to prevent drying. Ensure the top resin bed remains covered with DPBS. Never allow the column to run dry. Exposure of the resin to air may lead to channel formation and loss of separation performance.

### Sample loading and fraction collection

**Timing: Approximately 20–30 min per sample**

This step describes how to load clarified menstrual serum onto the equilibrated qEVoriginal 70 nm (Gen 2) column and manually collect size-based fractions containing small extracellular vesicles (sEVs).

15. Thaw the aliquoted menstrual serum completely on ice.
16. Remove the bottom luer cap to initiate gravity flow and allow any residual DPBS from the column flushing step to enter the resin bed. Wait until the column stops dripping and the liquid level reaches the upper frit.
17. Load **400 μL** sample volume directly onto the loading frit. Allow the sample to run into the column. Top up the column with particle-free DPBS to maintain a constant liquid level.
18. Immediately collect **400 μL fractions** sequentially into sterile, low-binding 1.5 mL microtubes (labelled F1-F10).
  a. Fractions 1–6 represent the column void volume and typically contain large particles and do not contain sEVs. These can be pooled and discarded.
  b. Fractions 7–10 correspond to the EV-enriched window, containing the majority of small extracellular vesicles. Collect these separately for downstream analyses.
19. Put F7-F10 on ice immediately after collection and store at −80°C for further analysis.

### Column cleaning and storage

**Timing: Approximately 40 min**

This section describes post-run cleaning and storage of the qEVoriginal 70 nm (Gen 2) column to maintain separation performance and prevent sample carry-over or microbial growth.

***Note:*** The precise fraction numbers corresponding to the EV-enriched window may vary slightly depending on sample viscosity, temperature, and column flow rate.

20. After the desired fractions have been collected, immediately clean the column to remove residual proteins and sample contaminants. Flush the column with **8.5 mL** of prepared 0.5 M NaOH solution to sanitize the resin and eliminate adsorbed biomolecules.
21. Follow by rinsing the column with **17 mL** of particle-free DPBS to restore the column pH to normal before loading another sample.

**CRITICAL:** Perform NaOH flushing immediately after use to prevent protein deposition.

Ensure that NaOH is fully displaced by particle-free DPBS before the next sample run. Simply washing with DPBS alone is not sufficient for complete decontamination; insufficient cleaning may result in carry-over between samples.

22. If storing the column for future use, first flush the column with two column volumes (17 mL) of particle-free deionized water, then rinse with two column volumes (17 mL) of particle-free 20% ethanol to avoid salt crystallization within the resin bed.
23. After cleaning and filling with the selected storage solution, seal the column tightly with both the top cap and luer slip cap.
24. Store the column at 4°C in a vertical position.

***Note:*** Each column can be reused up to five times, provided that the flow rate remains within specifications, and no visible contamination or flow resistance is observed.

### Characterization of MF-sEVs using nanoparticle tracking analysis

**Timing: Approximately 5 min per sample for acquisition**

This step quantifies particle concentration and size distribution of pooled EV fractions by NTA, which allows the capture and recording of particles moving under Brownian motion.^2^

***Note:*** The NTA (ZetaView, Germany, PMX-230-7) is used in this protocol. It is important to rinse the device of impurities before experimental samples are analyzed.

25. Turn on the NTA first and allow it to warm up for **20 min.**
26. Turn on the computer and open ZetaNavigator instrument software, and it will automatically check the components, wait until all indicators are green.
27. Click “Rinse” with water for 30 secs.
  a. If air bubbles are visible, pump them once again. If new water was added to the bottle, also rinse twice.
28. Prepare Size Standard Polystyrene (PS) at a 1/250000 dilution and inject PS by using 1 mL syringe and avoid introducing air bubbles, then click “Autofocus”.
  a. Yellow range is considered ok to continue. For autofocus 0 is the best, but 8 will still be green. You can save this and then re-run it to improve it.
29. Dilute EV samples with Particle-free DPBS (1 mL in total). Inject 1 mL sample and avoid introducing air bubbles, ensure that the particle number in diluted samples is between 10 to 100 per screen.
30. Analyze samples at 11 positions using the ZetaNavigator instrument software. Settings: laser 488 nm, sensitivity 82, shutter 100, sequence length 30, frame rate 30 and temperature 25°C.

***Note:*** Between samples, particle-free DPBS should be used to clean up to a particle number below 10 per screen.

31. Final cleaning: 20 mL particle-free dH2O, which can be repeated, until a particle number below 10 per screen.

*Important:* The value obtained from NTA represents the concentration of EVs per mL in the preprocessed menstrual fluid sample after removal of cells, debris, and impurities, and therefore reflects the EV concentration in the plasma fraction of the menstrual fluid. It should be noted that unlabeled NTA is not specific to extracellular vesicles, particularly in complex biofluids. Fluorescence-based NTA may be used as an optional approach to improve specificity, and results should be interpreted together with established EV marker analyses.

### Characterization of MF-sEVs using transmission electron microscopy

Samples were negatively stained for transmission electron microscopy (TEM) by applying 4 μl of sEV samples on glow-discharged, EM-Tec Formvar-carbon grids (200 mesh, Cu, Micro to Nano) and incubating for 1 min. Excess sample was blotted off with filter paper, then the sample was stained with 1% uranyl acetate solution and blotted to dry. Images were collected using a Talos 120C G2 (Thermo Fisher Scientific) equipped with a Ceta-D detector at 22,000× and 45,000× magnification.

### Characterization of MF-sEVs using western blotting

Western blotting was performed to validate the presence of canonical EV markers. EV-containing fractions (F7-F10) obtained from size-exclusion chromatography were pooled and concentrated using a centrifugal concentrator (Vivaspin 2, Sartorius, Göttingen, Germany; MWCO 10 kDa). Total protein was quantified using the Micro BCA Protein Assay Kit (Thermofisher, Cat.23235) following the manufacturer’s instructions. Fraction 1, which does not contain sEVs, was used as the negative control, positive control was total protein extracted from SH-SY5Y cell lysates. Equal amounts of protein (20 μg) were prepared and mixed with 5× reducing sample buffer and heated at 95°C for 5 min prior to SDS–PAGE. Proteins were separated on 10%–12% polyacrylamide gels and transferred onto PVDF membranes (0.22 μm pore size), which were activated by briefly soaking in methanol prior to transfer, using a semi-dry transfer system (Tanon 5200, Tanon, China) at 200 mA for 90 min. Membranes were blocked for 1 h in 5% skimmed milk prepared in PBST and incubated overnight at 4°C with the following primary antibodies: anti-CD9 (1:1000, SBI, EXOAB-CD9A-1), anti-CD63 (1:1000, SBI, EXOAB-CD63A-1), anti-CD81 (1:500–1:2000, Immunoway, YT5394), and anti-Calnexin (1:500–1:1000, Immunoway, YT0613). After washing, membranes were incubated with exosome-validated HRP-conjugated secondary antibody (Goat anti-Rabbit HRP, SBI, 1:20000) for 1 h at room temperature. Blots were developed using enhanced chemiluminescence (ECL) and imaged using the Tanon 5200 chemiluminescence detection system.

## Expected outcomes

This protocol yields a reproducible and high-purity population of small extracellular vesicles (sEVs) derived from menstrual fluid - a uniquely regenerative and non-invasive biofluid source that naturally undergoes cyclic tissue shedding and repair.^3^ Menstrual fluid contains abundant extracellular vesicles released from endometrial epithelia, stromal and immune cells, reflecting the dynamic molecular state of the uterine microenvironment during regeneration.^4^^,^^5^^,^^6^^,^^7^^,^^8^^,^^10^

**Figure 2:**
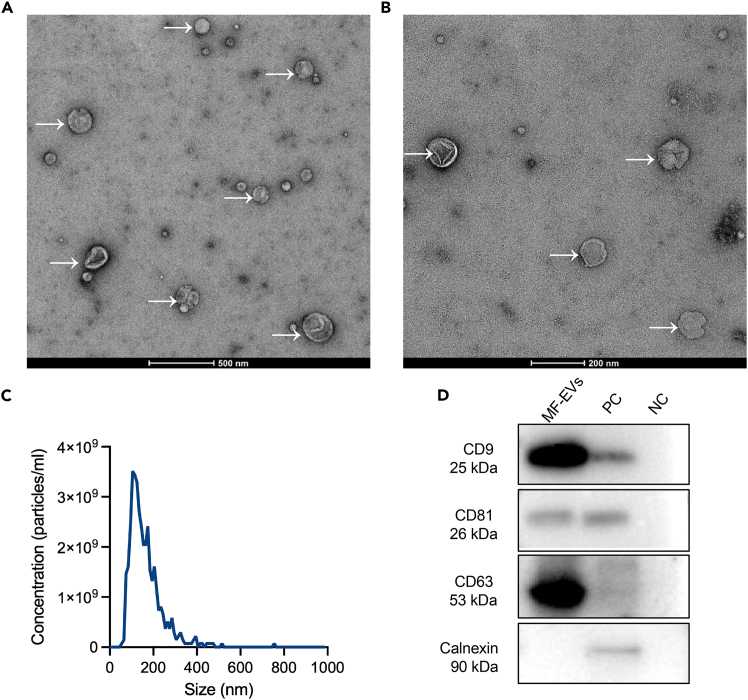
Characterization of MF-sEVs (A and B) Typical ultrastructure of MF-sEVs (white arrows) by transmission electron microscopy (TEM). Scale bars = 500 nm, 200 nm. (C) Representative nanoparticle tracking analysis (NTA) of MF-sEVs. (D) Western blot analysis of classical EV positive markers, including CD9, CD81, CD63, as well as the negative marker Calnexin.^3^ PC=positive control, NC=negative control.

The isolated vesicles typically appear as round or cup-shaped bilayered structures as observed by transmission electron microscopy (Figures 2A and 2B). Nanoparticle tracking analysis (NTA) shows a unimodal size distribution with a predominant peak around 100–150 nm. Western blot analysis confirmed successful enrichment of small extracellular vesicles from menstrual fluid. Robust bands corresponding to the classical EV markers CD63 (53 kDa), CD81 (26 kDa), and CD9 (25 kDa) were detected in MF-sEVs samples, consistent with the expected molecular weights (Figure 2D). Importantly, the endoplasmic reticulum protein Calnexin (90 kDa), a recommended negative control for non-vesicular contamination according to MISEV guidelines, was absent in MF-sEVs samples. This indicates minimal contamination from intracellular compartments and confirms the high purity of the isolated MF-sEVs.

Across independent isolations, the vesicle morphology, size distribution, and marker expression remain consistent, demonstrating the reproducibility of this SEC-based workflow. The resulting menstrual fluid-derived sEVs are suitable for downstream molecular profiling, regenerative modeling, and biomarker discovery.

## Limitations

This SEC-based workflow provides a reproducible and efficient method for isolating small extracellular vesicles (sEVs) from menstrual fluid. However, several limitations should be noted. First, the pre-clearance and 0.22 μm filtration steps effectively remove cells, clots, and large debris but also deplete larger extracellular vesicle subtypes such as microvesicles and apoptotic bodies. Therefore, this protocol preferentially enriches nanosized vesicles and does not capture the full vesicle spectrum. Second, menstrual fluid contains peripheral blood components and lipoproteins that may overlap in size with sEVs. Although size-exclusion chromatography separates vesicles from most soluble proteins and lipoproteins, complete elimination of non-vesicular particles cannot be guaranteed when working with complex biofluids. Users requiring stringent purity assessment may consider incorporating lipoprotein-specific assays (e.g., ApoA1/ApoB detection with ELISA or western blotting or fluorescence-based particle analysis) depending on downstream applications. Third, repeated reuse of SEC columns may alter flow rates, introduce cross-contamination, and reduce resolution between vesicular and protein fractions. For clinical or omics-scale studies, single-use columns or validated regeneration cycles with strict quality control are strongly recommended. Finally, NTA measures all light-scattering nanoparticles, including lipoproteins and protein aggregates, and cannot distinguish vesicular from non-vesicular particles without fluorescent labeling (e.g., f-NTA or antibody-coupled NTA).^9^ Similarly, BCA quantifies total soluble protein rather than EV-specific cargo; hence, protein-to-particle ratios should be interpreted as relative, not absolute, quality indicators.

## Troubleshooting

### Problem 1

Excess red blood cells, clots, or mucus in the menstrual fluid.

### Possible cause

Incomplete pre-filtration or hemolysis during collection.

### Potential solution

• Dilute the sample two to three times with particle-free DPBS in Step **6.**
• Pre-wet and perform sequential filtration through **100 μm** and **40 μm** cell strainers as described in Step **7** to remove clots and mucus completely.
• Avoid mechanical agitation and process samples promptly at **4°C** to minimize hemolysis.

### Problem 2

Low EV yield or weak NTA signal.

### Possible cause

Sample loss during handling or improper column operation.

### Potential solution

• Verify sample handling: minimize time from collection to processing; always keep samples on 4°C; avoid freeze–thaw before SEC.
• Limit pre-clear centrifugation to ≤2,000 × *g* for 10 min at 4°C in Step **9.**
• Load exactly 400 μL per qEVoriginal/70 nm run in Step **17**; avoid over/under-loading and collect the fractions accurately in Step **17.**
• Identify EV window per run: collect fractions 1–15 once, profile by NTA/BCA, then pool specific peak.
• If concentration is low, concentrate pooled fractions using a 10–50 kDa MWCO spin concentrator.

### Problem 3

High protein contamination in EV fractions (high BCA, poor purity).

### Possible cause

Incomplete column equilibration, viscous sample, or carry-over.

### Potential solution

• Equilibrate columns with ≥40 mL sterile DPBS before each run.
• Maintain steady drip flow and prevent the resin from drying during SEC in Step **18.**
• For viscous samples, pre-dilute 1.5–2× in DPBS.
• Narrow the fraction pooling window (fractions 7–8 only) or perform a second SEC pass.
• Validate purity by iodixanol gradient or immuno-capture on representative samples.
• Include process blanks; if blanks contain protein, replace buffers/consumables and recondition a new column.

## Resource availability

### Lead contact

Further information and requests for resources and reagents should be directed to and will be fulfilled by the lead contact, Yiqun Tang (yiqun.tang@ki.se).

### Technical contact

Technical inquiries regarding the protocol should be directed to the technical contact, Yiqun Tang (yiqun.tang@ki.se).

### Materials availability

This study did not generate new unique reagents.

### Data and code availability

This study did not generate new unique datasets or code.

## Acknowledgments

We thank the 3D-EM facility at Karolinska Institutet for access and technical support. Transmission electron microscopy (TEM) data were acquired at the 10.13039/501100004047Karolinska Institutet 3D-EM facility (https://ki.se/cmb/3d-em) with expert assistance from Amy Bondy. Western blotting guidance and positive control proteins extracted from SH-SY5Y cell lysates were kindly provided by Lisha Wang’s group at Karolinska Institutet. Nanoparticle tracking analysis (NTA) measurements were performed using an instrument generously made available by the Philip Newton group at Karolinska Institutet, and we are grateful for their technical support and assistance. The graphical abstract was created using BioRender.com under license.

## Author contributions

The study was conceptualized by Y.T. and K.G.-D. Experiments were conducted and figures/tables were prepared by Y.T. and R.Z. Ethical approval and sample collection were obtained and coordinated by C.F. and Y.T. The initial draft was written by Y.T., with critical review and revision by R.Z., C.F., and K.G.-D. K.G.-D. provided overall supervision. All authors read and approved the final version.

## Declaration of interests

The authors declare no competing interests.
